# Risk factors, prognostic factors, and nomograms for distant metastasis in patients with triple-negative breast cancer: A population-based study

**DOI:** 10.1097/MD.0000000000044770

**Published:** 2025-09-26

**Authors:** Hongguo Guo, Song Qiao, Cai Cheng, Jun Liu, Shangzhen Yang, Wanling Lu

**Affiliations:** a Department of Oncology, The 986th Hospital of the People’s Liberation Army Air Force, Xi’an, China.

**Keywords:** distant metastasis, nomograms, predictor, prognosis, triple-negative breast cancer

## Abstract

Triple-negative breast cancer (TNBC) has poor prognosis, high invasiveness, and limited treatments. This study analyzed TNBC patients’ clinical-pathological features, identified prognostic risk factors, and developed a predictive model to guide treatment and improve outcomes. We conducted a retrospective study on patients with TNBC from 2010 to 2015 in the surveillance, epidemiology, and end results (SEER) database. We collected tumor characteristics and treatment data from these patients. Univariate and multivariate logistic regression analyses were employed to identify independent risk factors for distant metastasis in TNBC patients, whereas univariate and multivariate Cox proportional hazards regression analyses were utilized to determine independent prognostic factors for TNBC patients with distant metastasis. On the basis of these factors, a new nomogram was constructed, and patients were categorized into high- and low-risk groups according to the nomogram scores. The prognosis of the patients was analyzed via Kaplan–Meier (K–M) survival analysis. This study analyzed 16,959 TNBC patients, with 4.3% presenting distant metastasis at diagnosis. Key risk factors included marital status, invasive lobular carcinoma, advanced T/N stage, no radiotherapy/surgery, and tumor size > 50mm. A nomogram with ROC curves (AUC: 0.892/0.907 training/validation sets) demonstrated excellent discrimination. For metastatic patients, unmarried status, no surgery/chemotherapy, and neoadjuvant therapy were independent prognostic factors (*P* < .001). The same results were obtained on the extended test set. Calibration curves, decision curve analysis (DCA), and K–M survival curves confirmed that both models could accurately predict the occurrence of distant metastasis and patient prognosis in patients with TNBC. We constructed 2 nomograms to predict distant metastasis risk in TNBC patients and prognosis in patients with distant metastasis. Our findings suggest that they could serve as effective predictive tools and may assist in clinical decision-making.

## 1. Introduction

Among female cancers, breast cancer has the highest mortality rate, with approximately 2.3 million new cases (11.7%) and 685,000 deaths (6.9%) reported in 2020.^[1]^ Triple-negative breast cancer (TNBC) is a unique breast cancer subtype characterized by a lack of expression of estrogen receptor (ER), progesterone receptor (PR), and human epidermal growth factor receptor 2 (HER2). Statistical data show that TNBC accounts for 10 to 20% of all breast cancer cases.^[2]^ TNBC displays complex biological characteristics, often resulting in increased tumor malignancy and a worse prognosis than other breast cancer types.

Because ER, PR and HER2 are not expressed, the prognosis is poor, there are few treatment options, and traditional targeted therapy and endocrine therapy are ineffective.^[3]^ Patients with TNBC usually rely only on conventional chemotherapy methods, and this treatment regimen may not be effective in controlling tumor progression. Studies have shown that, unlike other breast cancer variants, TNBC has higher 5-year mortality, is more aggressive, has a poorer prognosis, and reaches peak recurrence in the first 3 years after diagnosis. Distant metastasis occurs in approximately 50% of patients,^[4,5]^ and survival is even lower for patients with locally advanced or metastatic TNBC.

TNBC patients are more prone to distant metastasis, and early detection of distant metastasis through screening can improve survival rates.^[6]^ For many years, research on the correlation between traditional clinicopathological characteristics and distant metastasis in TNBC patients has been limited in scope. Existing studies primarily focus on metastatic tumor characteristics while neglecting a comprehensive understanding.^[7]^ Moreover, the prognosis of patients who have already experienced distant metastasis at the time of diagnosis requires further study. Risk factors associated with TNBC distant metastasis may identify high-risk patients, facilitating early clinical intervention.

In recent years, neoadjuvant therapy (NAT) has become a key component in the treatment of TNBC, offering surgical opportunities for late-stage patients and improving survival outcomes.^[8]^ For patients with inoperable or locally advanced breast cancer, the American Society of Clinical Oncology guidelines recommend the use of NATs.^[9]^ In addition to systemic therapy, a follow-up adjuvant therapy strategy has been developed on the basis of achieving pathological complete remission (pCR) at the primary site after the completion of the full course of NAT.^[10,11]^ However, the optimal NAT treatment regimen and duration of treatment remain a topic of debate,^[12]^ and further research in this area holds significant potential for advancing the treatment of TNBC.

Many years have passed since the American Joint Committee on Cancer (AJCC) prognostic staging system for tumors, lymph nodes, and metastasis (TNM) was first introduced to determine the prognosis of patients with breast cancer. With the development of molecular typing and precision therapies, the importance of traditional tumor staging methods has gradually decreased. Therefore, these data do not fully evaluate the possibility and future prospects of distant metastasis in TNBC patients.^[13,14]^ Due to the convenience and accuracy of nomograms, they have recently been widely used to evaluate the prognosis of cancer patients, which is a good choice.^[15,16]^ Therefore, in this study, a large number of TNBC patients were recruited for analysis via the surveillance, epidemiology, and end results (SEER) database. We investigated demographic characteristics (age, race, and marital status), tumor characteristics (histological type, grade, clinical T stage and N stage, and tumor size) and treatment methods (surgery, chemotherapy, radiotherapy and NAT) to assess the incidence, risk factors, and prognosis of TNBC metastasis and developed 2 nomograms to help clinicians make more appropriate medical decisions.

## 2. Materials and methods

### 2.1. Source of the data and patient selection

The study used a database called SEER, which was released in March 2024. Patients included in the study were selected from SEER * Stat version 8.4.3. The database contains population, patient characteristics, tumor characteristics, and diagnosis and healthcare information from 17 cancer registries, representing approximately 28% of cancer cases in the United States between 2000 and 2021.

We only utilized data from 2010 to 2015 because the SEER database exhibited inconsistencies in tumor T staging, N staging, and M staging information during subsequent periods (2016–2019 being a mixed registration phase of AJCC 7th and 8th editions, and 2020–2021 being AJCC 8th edition). Based on the following inclusion criteria, we analyzed breast cancer patients from January 1, 2010, to December 31, 2015: female 18 to 90 years old; initial diagnosis of primary breast cancer; and pathological analysis (ER-/PR-/HER 2-), confirming the triple-negative molecular subtype. Demographic variables, including age, sex and race, are available; Clinicopathological information, including primary tumor site, grade, histological type, TNM and tumor size, is available. The exclusion criteria were as follows: patients with multiple primary tumors and patients with missing or incomplete follow-up information. The data entered into the case table included age at diagnosis, ethnicity, marital status, histological type, grade, AJCC stage, tumor size, and cancer treatment. Relevant data factors were analyzed, and participants lacking clinical characteristics or survival information were excluded from the final dataset. Finally, this study included 16,959 TNBC patients, including 730 patients with distant metastases (patients clearly staged as M1 according to the AJCC 7th edition).

The age classification in this paper follows the 2023 United Nations World Health Organization guidelines, which are based on the assessment of global human body mass and average life expectancy. Accordingly, those aged 18 to 44 years were considered young; those aged 45 to 59 years were classified as middle-aged; and those aged over 60 years were classified as elderly. Many studies have established that morphologic evaluation of differentiation can provide valuable prognostic insights into breast cancer. Specifically, Grade I tumors were well differentiated, Grade II tumors were moderately differentiated, Grade III tumors were poorly differentiated, and Grade IV tumors were undifferentiated. For some anatomical sites, grades III and IV can be combined into a single level, and this classification is applicable for breast cancer. All patients were used to form a diagnostic cohort to explore risk factors for distant metastases and establish a predictive nomogram. Furthermore, specific treatment information, including surgery, chemotherapy, radiotherapy and NAT, can be used to form prognostic cohorts to study prognostic factors in patients with distant metastases and develop new prognostic nomograms.

Patients were divided into diagnostic and prognostic cohorts. In the diagnostic cohort, patients were randomly assigned to a training set (70%) or a validation set (30%), with a ratio of 7:3. The prognostic cohort was composed of patients with distant metastasis from both the training and validation sets of the diagnostic cohort. For each cohort, to prevent overfitting and ensure accuracy, nomograms were constructed using patients from the training set and validated using patients from the validation set.

### 2.2. Data collection

In this study, age, race, marital status, pathological type, grade, T stage, N stage, primary site surgery, radiotherapy, chemotherapy, tumor size and NAT were used as risk factors for distant metastasis in TNBC patients. Additionally, we conducted survival analyses with overall survival (OS) as the primary outcome to explore prognostic factors for patients with distant metastasis.

### 2.3. Statistical analysis

All studies were analyzed via R software (version 4.3.3), and *P* < .05 (two-sided) was considered statistically significant. All patients were randomly divided into training and validation sets, and the distributions of the 2 groups of variables were compared via the chi-square test or Fisher exact test.

In the diagnostic cohort, univariate logistic analysis was employed to identify risk factors associated with distant metastasis. Variables with *P* < .05 in the univariate analysis were subjected to multivariate logistic analysis to determine independent risk factors for distant metastasis in patients.^[17]^ Additionally, nomograms and receiver operating characteristic (ROC) curves were generated for all independent variables on the basis of individual risk factors, and the corresponding areas under the curve (AUC) was calculated to assess their discriminative ability. Additionally, calibration curves and decision curve analysis (DCA) were used to evaluate the performance of the nomograms.

For prognostic factors, we used univariate Cox regression analysis to identify factors associated with OS in patients with distant metastases. Subsequently, significant variables with *P* < .05 were subjected to “stepwise” multivariate Cox analysis to further determine independent prognostic factors. We constructed a prognostic nomogram based on these independent prognostic predictors to predict the overall survival of patients with distant metastases and calculated individual risk scores via the nomogram formula. Additionally, time-dependent ROC curves were generated for the nomogram and all independent prognostic variables at 12, 36, and 60 months, and the time-dependent AUC was used to evaluate the discriminative ability of the prognostic model. Calibration curves and DCA were plotted at 12, 36, and 60 months to assess the flexibility and clinical applicability of the predictive model. All patients with distant metastases were divided into high-risk and low-risk groups on the basis of the median risk score. Kaplan–Meier (K–M) survival curves and log-rank tests were used to demonstrate the difference in overall survival status between the 2 groups.

The aforementioned statistical analysis utilized various R packages, such as “Survival,” “ ggplot,” “Table 1,” “rms,” “ stdca. R,” “survivalROC,” and “ survivalminer” (http://www.r-project.org/).

**Table 1:** Baseline clinical characteristics of TNBC patients.

	Training	Validation	*χ* ^2^	*P*	Overall
	(N = 11,871)	(N = 5088)			(N = 16,959)
Age group			1.472	.479	
>60	4670 (39.3%)	2052 (40.3%)			6722 (39.6%)
20–44	2382 (20.1%)	1001 (19.7%)			3383 (19.9%)
45–59	4819 (40.6%)	2035 (40.0%)			6854 (40.4%)
Race			2.078	.556	
AI/AN	76 (0.6%)	26 (0.5%)			102 (0.6%)
Asian	932 (7.9%)	423 (8.3%)			1355 (8.0%)
Black	2164 (18.2%)	934 (18.4%)			3098 (18.3%)
White	8699 (73.3%)	3705 (72.8%)			12,404 (73.1%)
Marital			2.045	.153	
Married	9002 (75.8%)	3911 (76.9%)			12,913 (76.1%)
Not married	2869 (24.2%)	1177 (23.1%)			4046 (23.9%)
Histology			2.830	.243	
IDC	10,244 (86.3%)	4419 (86.9%)			14,663 (86.5%)
ILC	310 (2.6%)	111 (2.2%)			421 (2.5%)
Other	1317 (11.1%)	558 (11.0%)			1875 (11.1%)
Grade			0.168	.920	
I	254 (2.1%)	105 (2.1%)			359 (2.1%)
II	1987 (16.7%)	844 (16.6%)			2831 (16.7%)
III–IV	9630 (81.1%)	4139 (81.3%)			13,769 (81.2%)
**T**			0.593	.898	
T1	5273 (44.4%)	2290 (45.0%)			7563 (44.6%)
T2	5049 (42.5%)	2133 (41.9%)			7182 (42.3%)
T3	975 (8.2%)	418 (8.2%)			1393 (8.2%)
T4	574 (4.8%)	247 (4.9%)			821 (4.8%)
N			1.161	.763	
N0	7767 (65.4%)	3314 (65.1%)			11,081 (65.3%)
N1	2854 (24.0%)	1257 (24.7%)			4111 (24.2%)
N2	707 (6.0%)	293 (5.8%)			1000 (5.9%)
N3	543 (4.6%)	224 (4.4%)			767 (4.5%)
Surgery prim			3.926	.140	
Breast-conserving	5402 (45.5%)	2386 (46.9%)			7788 (45.9%)
Mastectomy	5749 (48.4%)	2380 (46.8%)			8129 (47.9%)
No	720 (6.1%)	322 (6.3%)			1042 (6.1%)
Radiation			0.107	.744	
No	5657 (47.7%)	2410 (47.4%)			8067 (47.6%)
Yes	6214 (52.3%)	2678 (52.6%)			8892 (52.4%)
Chemotherapy			0.487	.485	
No	2595 (21.9%)	1087 (21.4%)			3682 (21.7%)
Yes	9276 (78.1%)	4001 (78.6%)			13,277 (78.3%)
Tumor size			2.219	.330	
<50	10,580 (89.1%)	4541 (89.2%)			15,121 (89.2%)
>100	197 (1.7%)	69 (1.4%)			266 (1.6%)
50–100	1094 (9.2%)	478 (9.4%)			1572 (9.3%)
Neoadjuvant therapy			6.567	.161	
CR	633 (5.3%)	291 (5.7%)			924 (5.4%)
No^*^	10,033 (84.5%)	4320 (84.9%)			14,353 (84.6%)
Not noted	481 (4.1%)	167 (3.3%)			648 (3.8%)
NR	139 (1.2%)	60 (1.2%)			199 (1.2%)
PR	585 (4.9%)	250 (4.9%)			835 (4.9%)

CR = complete remission, IDC = invasive ductal carcinoma, ILC = invasive lobular carcinoma, No^*^ = neoadjuvant therapy not given, Not noted = response to treatment but not noted if complete or partial, NR = no remission, PR = partial remission.

## 3. Results

### 3.1. Baseline characteristics of the study population

A total of 16,959 TNBC patients who met the criteria were included from the SEER database, of which 11,871 patients were included in the training set and 5088 patients were included in the validation set. The average ages for the training and validation sets were 55.95 years (ranging from 20–90 years) and 56.16 years (ranging from 21–90 years), respectively. The most common pathological type was invasive ductal carcinoma (86.3% for the training set and 86.9% for the validation set), and the most common tumor differentiation grade was III to IV (81.1% for the training set and 81.3% for the validation set). The most common T and N stages were T1 (44.4% for the training set and 45.0% for the validation set) and N0 (65.4% for the training set and 65.1% for the validation set), respectively. Overall, no significant differences were found between the training and validation sets (*P* > .05) (Table 1).

### 3.2. Incidence and risk factors for distant metastasis in patients with TNBC

At the initial diagnosis, 730 patients (4.3%) were identified with distant metastasis, whereas 16,229 patients (95.7%) were found to be free of distant metastasis. Table 2 presents the results of a univariate logistic regression analysis conducted on 12 potential factors, revealing that 9 variables are significantly associated with distant metastasis. These variables include marital status, pathological type, T stage, N stage, primary site surgery, radiotherapy, chemotherapy, tumor size and NAT. Furthermore, multivariate logistic regression analysis revealed several independent risk factors for distant metastasis in TNBC patients: being married, pathological invasive lobular carcinoma, high T stage, high N stage, no radiotherapy, no surgery, and a tumor size exceeding 50 mm (Table 2).

**Table 2:** Univariate and multivariate logistic analyses of distant metastasis in TNBC patients.

	Univariate analysis			Multivariate analysis		
	OR	95% CI	*P*	OR	95% CI	*P*
Age group						
20–44	Reference					
45–59	0.965	0.814–1.148	.735			
>60	1.065	0.900–1.265	.540			
Race						
White	Reference					
Black	1.165	0.994–1.359	.107			
Asian	0.932	0.726–1.179	.633			
AI/AN	1.949	0.999–3.428	.072			
Marital						
Married	Reference			Reference		
Not married	1.457	1.271–1.666	<.001	0.814	0.690–0.958	.039
Histology						
IDC	Reference			Reference		
ILC	2.350	1.743–3.105	<.001	1.843	1.298–2.571	0.003
Other	1.306	1.083–1.565	.017	1.017	0.807–1.272	0.900
Grade						
I	Reference					
II	1.617	0.945–3.028	.171			
III–IV	1.797	1.073–3.315	.085			
T						
T1	Reference			Reference		
T2	2.576	2.121–3.145	<.001	1.554	1.262–1.923	<.001
T3	8.783	7.069–10.941	<.001	1.774	1.191–2.632	.017
T4	30.340	24.706–37.435	<.001	5.044	3.671–6.916	<.001
N						
N0	Reference			Reference		
N1	5.873	4.995–6.925	<.001	3.021	2.511–3.638	<.001
N2	6.714	5.342–8.403	<.001	4.252	3.261–5.520	<.001
N3	19.071	15.680–23.205	<.001	8.012	6.321–10.155	<.001
Surgery prim						
Breast-conserving	Reference			Reference		
Mastectomy	2.776	2.288–3.388	<.001	1.142	0.924–1.420	.305
No	41.499	34.098–50.820	<.001	13.636	10.824–17.259	<.001
Radiation						
No	Reference			Reference		
Yes	0.493	0.433–0.561	<.001	0.633	0.537–0.746	<.001
Chemotherapy						
No	Reference					
Yes	1.400	1.189–1.658	.001			
Tumor size						
<50	Reference			Reference		
>100	13.865	10.898–17.533	<.001	2.343	1.607–3.417	<.001
50–100	5.941	5.147–6.847	<.001	1.803	1.305–2.503	.003
Neoadjuvant therapy						
CR	Reference					
No^*^	1.487	1.086–2.099	.046			
Not noted	1.669	1.073–2.605	.056			
NR	2.708	1.544–4.631	.002			
PR	1.759	1.168–2.679	.024			

CR = complete remission, IDC = invasive ductal carcinoma, ILC = invasive lobular carcinoma, No^*^ = neoadjuvant therapy not given, Not noted = response to treatment but not noted if complete or partial, NR = no remission, PR = partial remission.

### 3.3. Nomogram and validation of the TNBC diagnostic cohort

**Figure 1.**
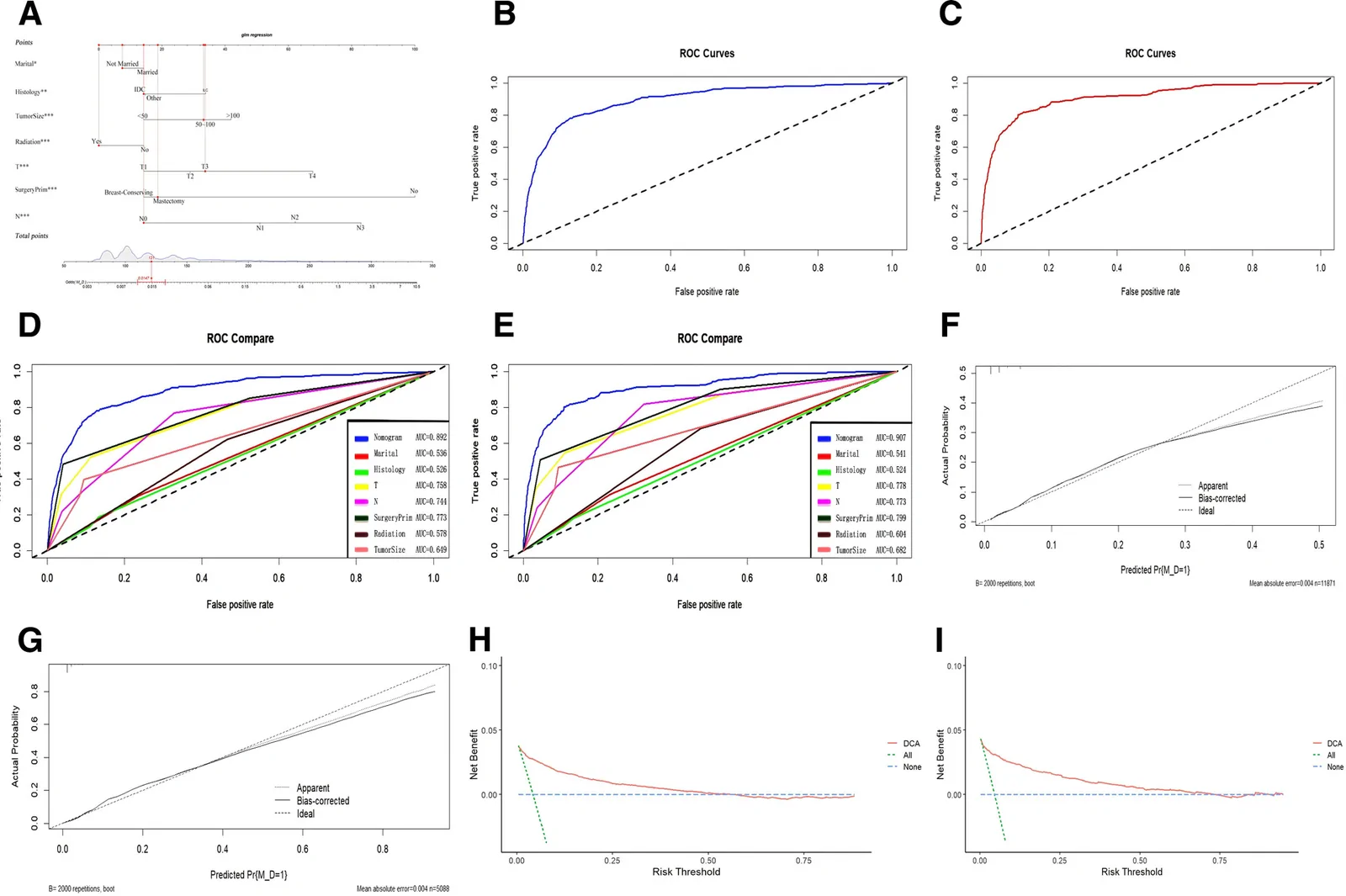
(A) Nomogram for assessing the risk of distant metastasis in TNBC patients. (B) ROC curve for the training set AUC = 0.892. (C) ROC curve for the validation set AUC = 0.907. (D) Comparison of the areas under the receiver operating characteristic curves for various factors in the training set. (E) Comparison of the areas under the receiver operating characteristic curves for various factors in the validation set. (F) Calibration curve of the training set. (G) Calibration curve of the validation set. (H) DCA curve of the training set. (I) DCA curves for the validation set. AUC = areas under the curves, DCA = decision curve analysis, ROC = receiver operating characteristic, TNBC = triple-negative breast cancer.

On the basis of these 7 independent predictive factors, a nomogram was developed to predict the risk of distant metastasis in patients with TNBC (Fig. 1A). To validate the performance of the nomogram, ROC curves were plotted for both the training and validation sets, with AUCs of 0.892 and 0.907, respectively (Fig. 1B and C). To verify its predictive ability, ROC curves for all the independent predictive factors were plotted (Fig. 1D and E), which revealed that their discriminative ability in the training and validation sets was superior to that of the other single factors. Moreover, the calibration curves drawn showed good consistency between the observed outcomes and the predicted outcomes (Fig. 1F and G). By examining the DCA curves, the net benefit of using the scale as a tool to trigger medical intervention compared with comprehensive treatment or no treatment was demonstrated, allowing us to decide whether using the model to assist in clinical decision-making would improve the treatment outcomes of our patients (Fig. 1H and I).

### 3.4. Prognostic factors of TNBC patients with distant metastasis

In this study, a cohort of 730 eligible patients with distant metastasis was assembled to investigate prognostic factors. As shown in Table 3, surgical intervention was performed on 372 patients (51.0%), radiotherapy was performed on 262 patients (35.9%), and chemotherapy was administered to 608 patients (83.3%). Both the χ^2^ test and Fisher exact test demonstrated no statistically significant differences across all variables between the training and validation sets (*P* > .05). Furthermore, univariate and multivariate prognostic regression analyses revealed that being unmarried (*P* < .001), not undergoing surgery (*P* < .001), not receiving chemotherapy (*P* < .001) and having NAT results emerged as independent prognostic factors for TNBC patients with distant metastasis, as outlined in Table 4.

**Table 3:** Baseline clinical characteristics of patients diagnosed with TNBC accompanied by distant metastasis.

	Training	Validation	*χ* ^2^	*P*	Overall
	(N = 504)	(N = 226)			(N = 730)
Age group			0.462	.793	
20–44	89 (17.7%)	55 (24.3%)			144 (19.7%)
45–59	197 (39.1%)	85 (37.6%)			282 (38.6%)
>60	218 (43.3%)	86 (38.1%)			304 (41.6%)
Race			0.629	.889	
White	354 (70.2%)	165 (73.0%)			519 (71.1%)
Black	104 (20.6%)	46 (20.4%)			150 (20.5%)
Asian	39 (7.7%)	14 (6.2%)			53 (7.3%)
AI/AN	7 (1.4%)	1 (0.4%)			8 (1.1%)
Marital			0.003	.959	
Married	342 (67.9%)	162 (71.7%)			504 (69.0%)
Not married	162 (32.1%)	64 (28.3%)			226 (31.0%)
Histology			0.539	.763	
IDC	406 (80.6%)	188 (83.2%)			594 (81.4%)
ILC	27 (5.4%)	11 (4.9%)			38 (5.2%)
Other	71 (14.1%)	27 (11.9%)			98 (13.4%)
Grade			0.299	.861	
I	5 (1.0%)	4 (1.8%)			9 (1.2%)
II	81 (16.1%)	32 (14.2%)			113 (15.5%)
III–IV	418 (82.9%)	190 (84.1%)			608 (83.3%)
T			1.112	.774	
T1	66 (13.1%)	35 (15.5%)			101 (13.8%)
T2	163 (32.3%)	79 (35.0%)			242 (33.2%)
T3	106 (21.0%)	42 (18.6%)			148 (20.3%)
T4	169 (33.5%)	70 (31.0%)			239 (32.7%)
N			2.156	.541	
N0	113 (22.4%)	44 (19.5%)			157 (21.5%)
N1	223 (44.2%)	97 (42.9%)			320 (43.8%)
N2	60 (11.9%)	28 (12.4%)			88 (12.1%)
N3	108 (21.4%)	57 (25.2%)			165 (22.6%)
Surgery prim			3.150	.207	
Breast-conserving	65 (12.9%)	32 (14.2%)			97 (13.3%)
Mastectomy	191 (37.9%)	84 (37.2%)			275 (37.7%)
No	248 (49.2%)	110 (48.7%)			358 (49.0%)
Radiation			2.258	.132	
No	327 (64.9%)	141 (62.4%)			468 (64.1%)
Yes	177 (35.1%)	85 (37.6%)			262 (35.9%)
Chemotherapy			0.193	.659	
No	80 (15.9%)	42 (18.6%)			122 (16.7%)
Yes	424 (84.1%)	184 (81.4%)			608 (83.3%)
Tumor size			3.088	.213	
<50	282 (56.0%)	142 (62.8%)			424 (58.1%)
>100	56 (11.1%)	20 (8.8%)			76 (10.4%)
50–100	166 (32.9%)	64 (28.3%)			230 (31.5%)
Neoadjuvant therapy			2.409	.661	
CR	18 (3.6%)	9 (4.0%)			27 (3.7%)
No^*^	427 (84.7%)	188 (83.2%)			615 (84.2%)
Not noted	23 (4.6%)	8 (3.5%)			31 (4.2%)
NR	11 (2.2%)	4 (1.8%)			15 (2.1%)
PR	25 (5.0%)	17 (7.5%)			42 (5.8%)

CR = complete remission, IDC = invasive ductal carcinoma, ILC = invasive lobular carcinoma, No^*^ = neoadjuvant therapy not given, Not noted = response to treatment but not noted if complete or partial, NR = no remission, PR = partial remission.

**Table 4:** Univariate and multivariate Cox analyses of patients with distant metastasis of TNBC.

	Univariate analysis			Multivariate analysis		
	HR	95% CI	*P*	HR	95% CI	*P*
Age group						
20–44	Reference					
45–59	1.107	0.922–1.328	.358			
>60	1.238	1.035–1.481	.049			
Race						
White	Reference					
Black	1.025	0.872–1.204	.800			
Asian	0.923	0.712–1.197	.615			
AI/AN	1.067	0.571–1.998	.863			
Marital						
Married	Reference			Reference		
Not married	1.469	1.278–1.688	<.001	1.390	1.207–1.599	<.001
Histology						
IDC	Reference					
ILC	0.827	0.615–1.112	.292			
Other	0.922	0.759–1.121	.496			
Grade						
I	Reference					
II	1.217	0.665–2.228	.592			
III–IV	1.126	0.626–2.024	.738			
T						
T1	Reference					
T2	0.944	0.767–1.162	.652			
T3	1.066	0.851–1.336	.640			
T4	1.154	0.937–1.420	.256			
N						
N0	Reference					
N1	1.437	1.206–1.712	.001			
N2	1.298	1.025–1.643	.068			
N3	1.150	0.941–1.405	.249			
surgery prim						
Breast-conserving	Reference			Reference		
Mastectomy	0.815	0.661–1.005	.109	0.815	0.661–1.005	.109
No	1.754	1.435–2.143	<.001	1.689	1.381–2.066	<.001
NonPrimSurg						
No	Reference					
Yes	0.642	0.493–0.835	.006			
Radiation						
No	Reference					
Yes	0.755	0.658–0.866	.001			
Chemotherapy						
No	Reference			Reference		
Yes	0.508	0.427–0.602	<.001	0.502	0.423–0.596	<.001
Tumor size						
<50	Reference					
>100	1.136	0.913–1.413	.336			
50–100	1.072	0.929–1.237	.423			
Neoadjuvant therapy						
CR	Reference					
No^*^	5.776	3.412–9.774	<.001	3.718	2.178–6.348	<.001
Not noted	4.071	2.218–7.468	<.001	3.894	2.121–7.147	<.001
NR	7.184	3.661–14.097	<.001	7.058	3.592–13.866	<.001
PR	2.564	1.405–4.676	.001	2.465	1.351–4.502	.013

CR = complete remission, IDC = invasive ductal carcinoma, ILC = invasive lobular carcinoma, No^*^ = neoadjuvant therapy not given, Not noted = response to treatment but not noted if complete or partial, NR = no remission, PR = partial remission.

### 3.5. Prognostic nomogram and validation

**Figure 2.**
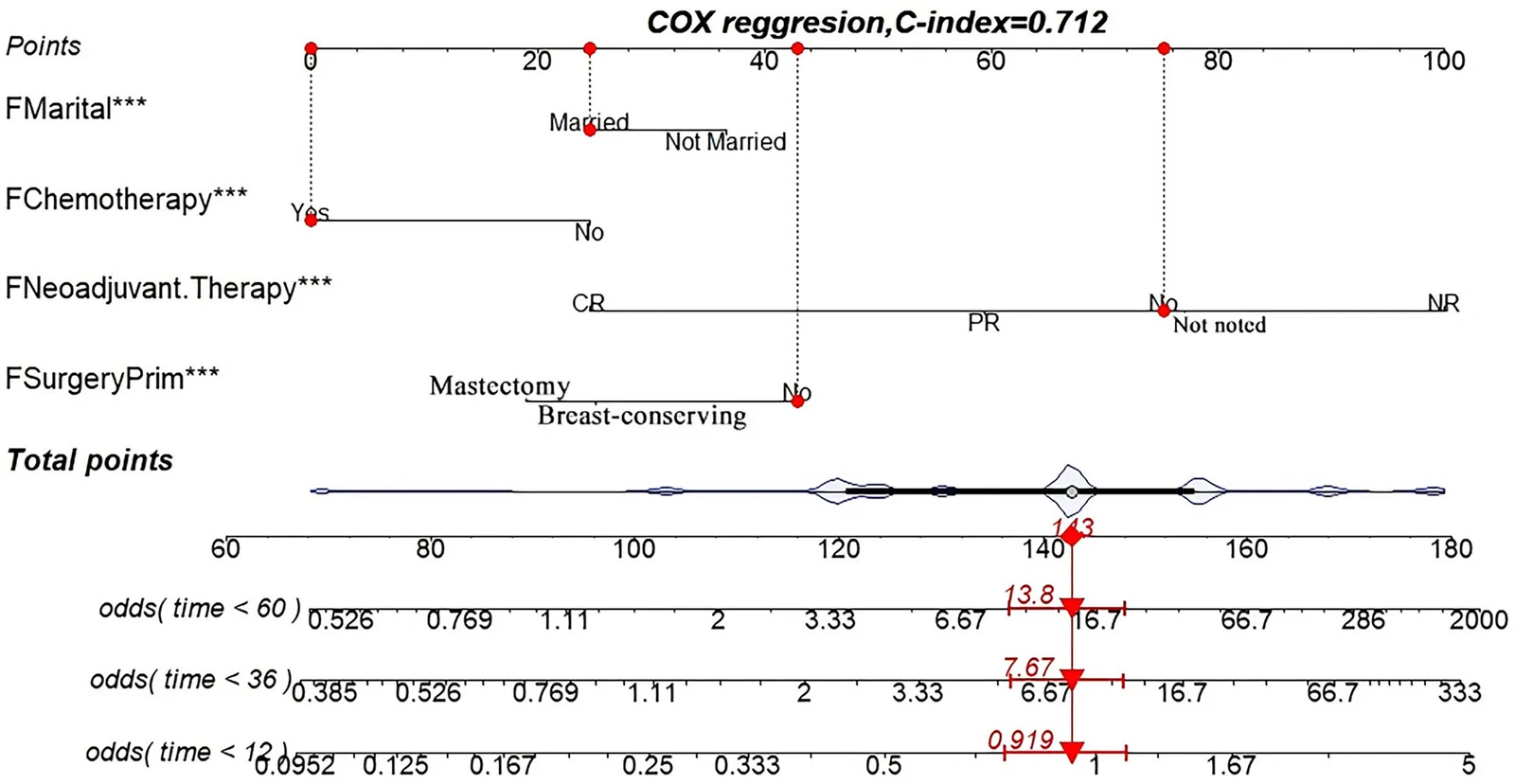
Prognostic nomogram for predicting OS in TNBC patients with distant metastasis at 12, 36, and 60 mo. OS = overall survival, TNBC = triple-negative breast cancer.

**Figure 3.**
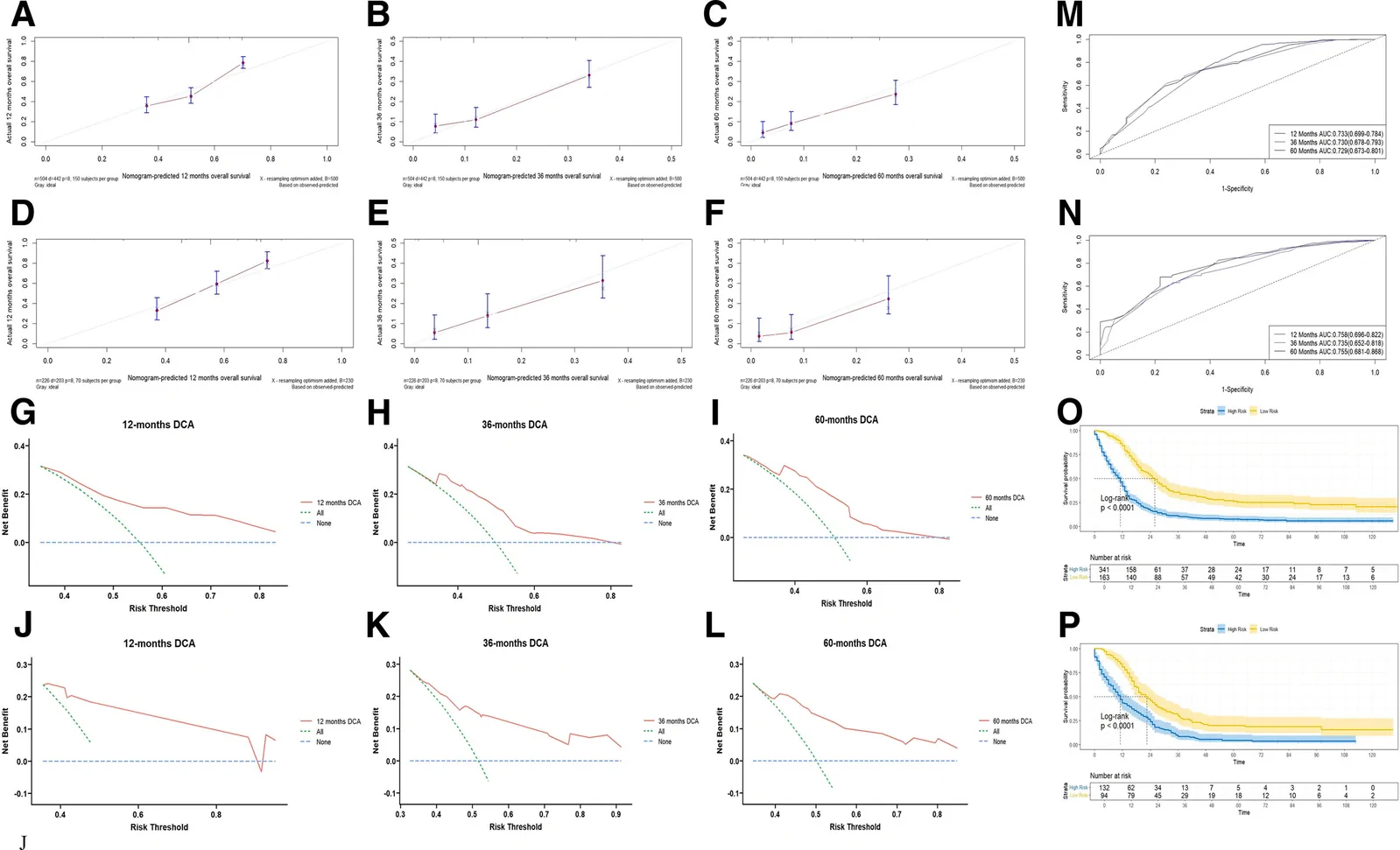
(A–C) Training set calibration curve: 12, 36, 60 mo. (D–F) Validation set calibration curve: 12, 36, 60 mo. (G–I) Training set DCA curve: 12, 36, 60 mo. (J–L) Validation set DCA curve: 12, 36, 60 mo. (M) Training set ROC curves: 12, 36, and 60 mo. (N) Validation set ROC curves: 12, 36, and 60 mo. (O) OS of the high-risk and low-risk groups in the training set. (P) OS of the high-risk and low-risk groups in the validation set. DCA = decision curve analysis, OS = overall survival, ROC = receiver operating characteristic.

**Figure 4.**
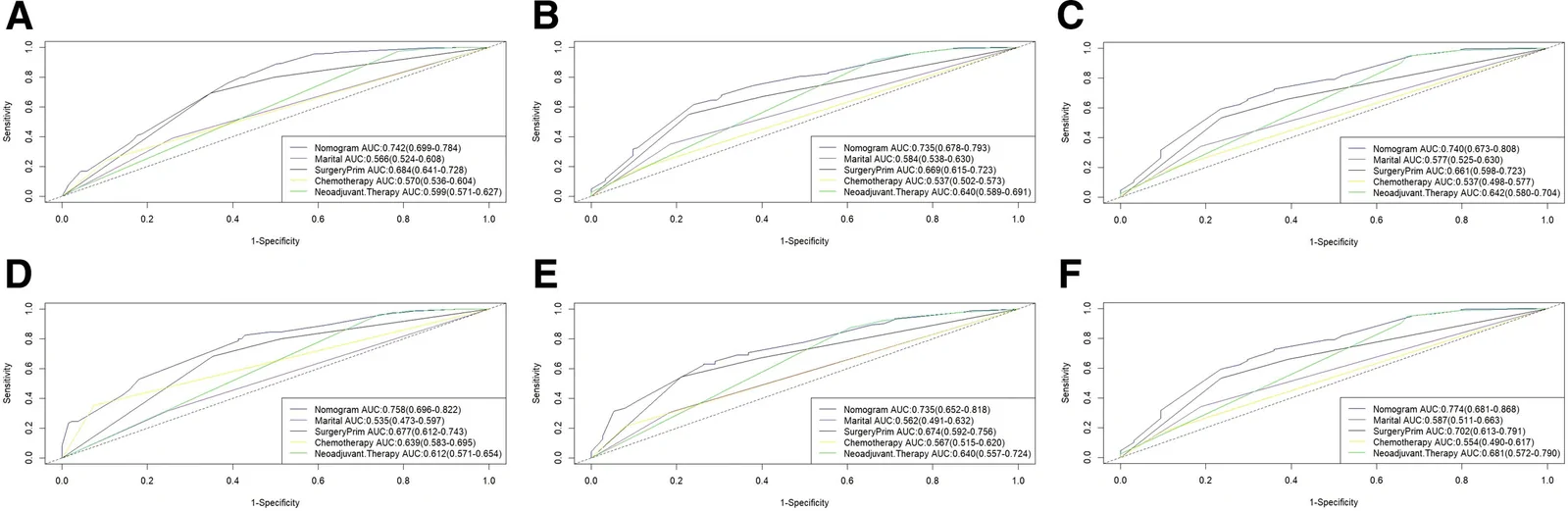
(A–C) Comparison of the area under the receiver operating characteristic curve for the 12-, 36-, and 60-mo training sets. (D–F) Comparison of the area under the receiver operating characteristic curve for the 12-, 36-, and 60-mo validation set.

Based on 4 prognostic factors, including unmarried (*P* < .001), not undergoing surgery (*P* < .001), not receiving chemotherapy (*P* < .001) and NAT results, we constructed a nomogram to predict the OS of patients with distant metastases (Fig. 2). Calibration curves were plotted separately in the training set (Fig. 3A–C) and validation set (Fig. 3D–F), which revealed strong consistency between the predicted OS probabilities at 12, 36, and 60 months and the actual outcomes. In the DCA curve, the horizontal coordinate axis (y = 0) represents “intervention for all patients,” the straight line starting from the origin with a negative slope represents “no intervention for all patients,” and the nomogram model curve lies above both the “intervention for all patients” and “no intervention for all patients” curves, indicating that this nomogram demonstrates good performance in clinical practice (Fig. 3G–L). The ROCs in the training set were 0.733, 0.730, and 0.729 at 12, 36, and 60 months, respectively, whereas the ROCs in the validation set were 0.758, 0.735, and 0.755, respectively (Fig. 3M and N). Finally, each patient was assigned a risk score on the basis of the 4 features of the nomogram, and a risk classification was developed on this basis. The K–M curves revealed that the OS of patients in the high-risk group was significantly lower than that of patients in the low-risk group (Fig. 3O and P). Moreover, by comparing the nomogram with each independent prognostic factor at 12, 36, and 60 months, the nomogram outperformed all the independent prognostic factors (Fig. 4A–F).

### 3.6. Validation of the performance of the nomogram in an extended test set

**Figure 5.**
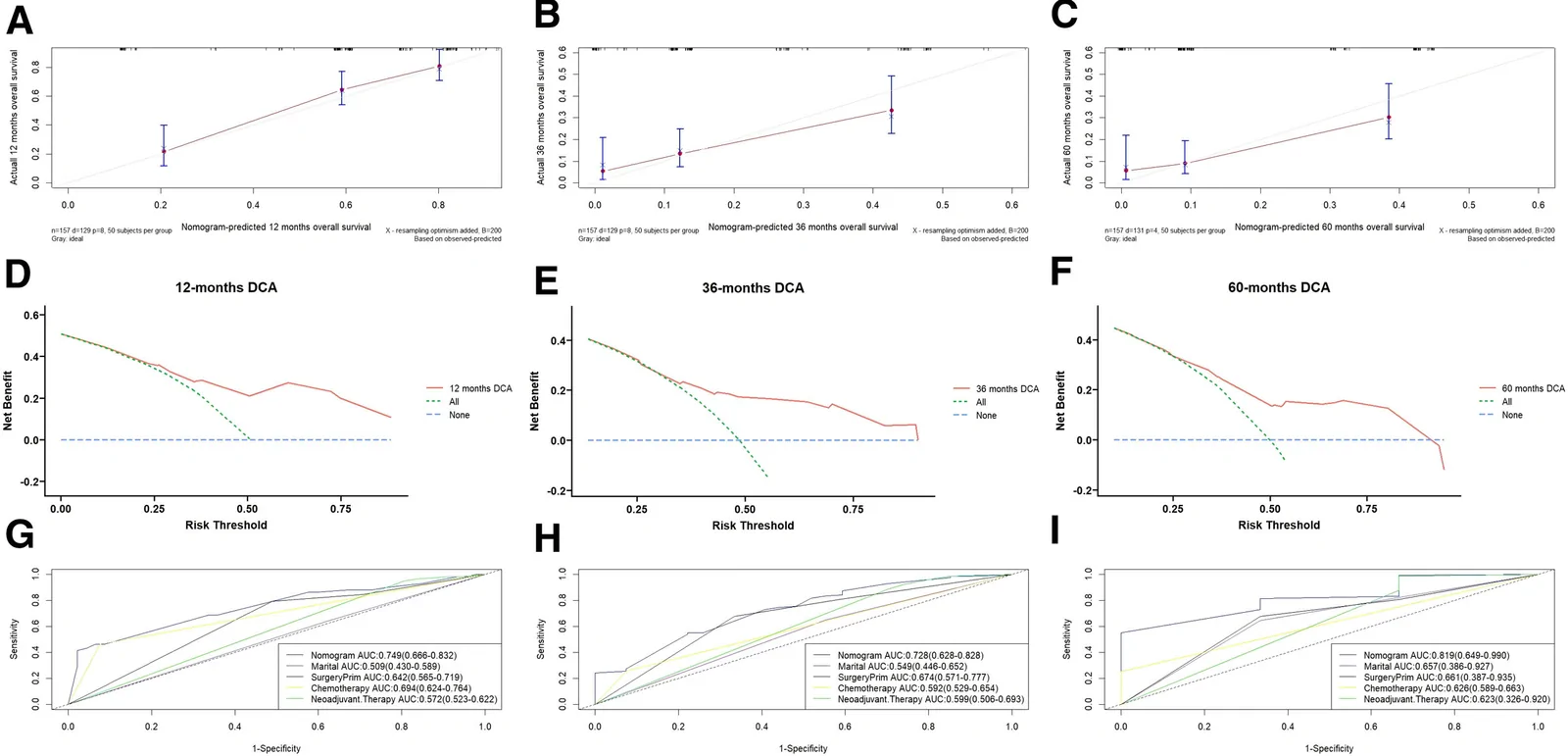
(A–C) Extended set calibration curve: 12, 36, 60 mo. (D–F) Extended set DCA curve: 12, 36, 60 mo. (G–I) Comparison of the area under the receiver operating characteristic curve for the 12-, 36-, and 60-mo extended set. DCA = decision curve analysis.

**Figure 6.**
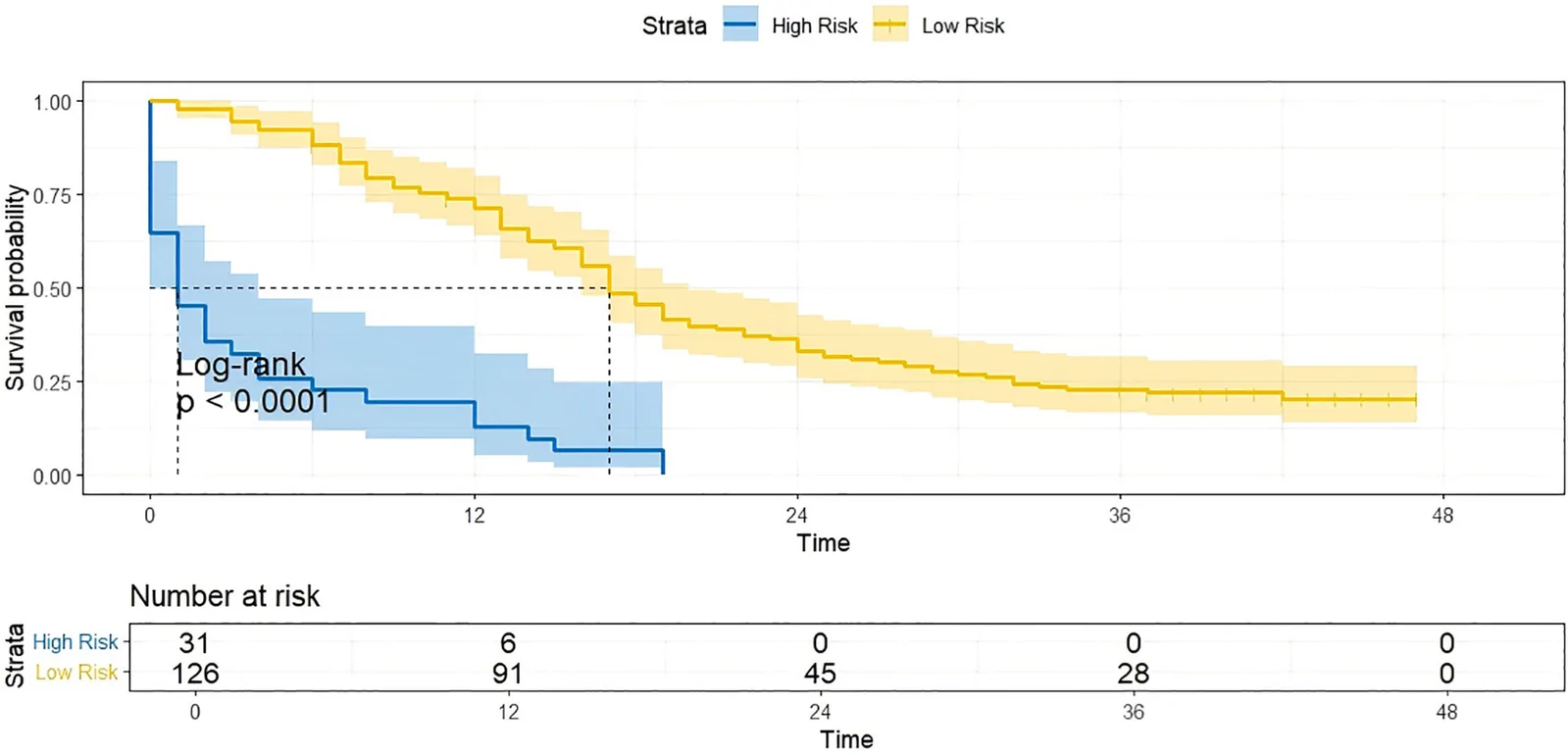
OS of high-risk and low-risk patients in the extended set.

An expanded test set was established by rescreening the SEER database to identify 157 TNBC patients diagnosed with distant metastasis in 2017, all with comprehensive information regarding marital status, chemotherapy, surgery and NAT results. Within this expanded test set, the calibration curve of the prognostic nomogram demonstrated a high degree of concordance between the predicted overall survival of patients with distant metastasis and the actual observed outcomes (Fig. 5A–C). Furthermore, the prognostic nomogram emerged as an effective clinical tool, as evidenced by the DCA results (Fig. 5D–F). Notably, at 12, 36, and 60 months, the performance of the nomogram surpassed that of the 4 independent predictors (Fig. 5G–I). Additionally, the AUCs for overall survival at 12, 36, and 60 months were 0.749, 0.728, and 0.819, respectively. K–M survival analysis revealed significant differences in survival patterns between patients categorized as high-risk and low-risk (*P* < .05) (Fig. 6).

## 4. Discussion

TNBC is a type of breast cancer with unique biological characteristics and clinical manifestations that is prone to distant metastasis.^[18]^ Owing to the diversity and heterogeneity of TNBC, treatment strategies should be adjusted according to patients’ physiological and clinical characteristics of patients. In this study, we performed an in-depth analysis of risk factors for distant metastasis and post-metastasis prognosis. Through statistical analysis of the clinical data, we identified multiple factors significantly affecting distant metastasis risk and overall prognosis. In this study, 16,959 eligible TNBC patients were screened through the SEER database. Subsequent analyses employed both univariate and multivariate logistic regression to identify factors associated with distant metastasis, along with Cox regression for prognostic evaluation. The results demonstrated that marital status, T stage, N stage, surgery, radiotherapy, and tumor size were independent risk factors for distant metastasis in patients with TNBC. Marital status, surgery, chemotherapy and NAT are significant predictors of OS in patients with distant metastasis of TNBC. These independent risk variables were mostly consistent with clinical observations, and we constructed a diagnostic nomogram to predict distant metastasis in TNBC patients and a prognostic nomogram for such patients. Relevant scores can be calculated by obtaining data from several key accessible variables on the nomogram, while our nomogram has strong risk classification ability for TNBC patients and can be applied to patient survival information, as well as direct clinical decision-making and treatment allocation. We recommend classifying high-risk patients as high-risk patients according to the tumor map, providing intensive care and thorough follow-up due to poor prognosis, and overall, using this model, investigators can reduce patient psychological distress, improve treatment and follow-up compliance, and collect more valuable data to design clinical trials.

In recent years, although similar work has been performed by other researchers,^[19–21]^ most studies have focused on molecular mechanisms rather than clinicopathological features‌. In this study, we combined the latest large sample and comprehensive clinical information from the SEER database and reported that the incidence of distant metastasis was 4.3%, which was lower than that reported in previous studies. We identified 7 important predictors of distant metastasis in triple-negative breast cancer (TNBC) patients, namely, marital status, pathological type, N stage, T stage, radiotherapy, surgery and tumor size. Multiple prior reports have consistently demonstrated that younger age is closely linked to distant metastasis and an unfavorable prognosis. A study encompassing 143 TNBC patients revealed that individuals aged 35 years or younger presented a 2.71-fold greater risk of relapse-free survival (RFS) and a 2.08-fold greater risk of distant relapse-free survival than did those over 50 years of age.^[22]^ Another study enrolled 1930 TNBC patients, with 15% being diagnosed at an age below 40 years and the remaining 85% being 40 years or older. With a median follow-up duration of 74 months, no significant disparities were observed in terms of local recurrence or disease-free survival among the different age groups. Multivariate analysis further revealed that larger tumor size, lymphovascular invasion, and positive lymph node status were significant predictors of increased disease recurrence risk, whereas age and surgical approach did not exhibit any notable associations with either outcome.^[23]^ Furthermore, in another report with 254 patients with TNBC, 75.6% of the patients were younger than 65 years old, and 24.4% were aged 65 years or older. The results revealed that the incidence of distant visceral metastases was significantly greater than that of bone metastases in both age groups (*P* < .001). A significantly greater number of local recurrences, bone metastases, and secondary lymph node metastases were observed in younger patients (*P* = .035, 0.025, and 0.041, respectively).^[24]^ In contrast, our findings indicate that age is not an independent risk factor for distant metastasis in TNBC patients. The discrepancies between studies may be attributed to the following factors. First, our study specifically targeted TNBC, unlike previous research that included other breast cancer subtypes. Second, by leveraging data from the population-based SEER registry, our analysis benefits from a larger and more representative TNBC cohort compared to single-center studies, resulting in findings with broader generalizability to the general population.

This study identified marital status as an independent risk factor for distant metastasis and the prognosis of primary TNBC patients. Some previous reports suggest that separated and widowed white women have a greater risk of death than married white women do and that the same is true for single and divorced white women. For Asian/Pacific Islanders (APIs), only single and divorced women had a higher risk of death than married women did. Marital status did not impact the risk of death among Black or Hispanic women. The research findings indicate: The risk of mortality associated with marital status is dependent on race/ethnicity. Only white and API women with TNBC have a marital advantage.^[25]^ In the construction of another predictive model, patients with better survival outcomes were unmarried, uninsured, patients with higher T and N stages, and patients with a histological type of mixed invasive ductal carcinoma and lobular carcinoma. The prognosis of eTNBC patients is influenced mainly by marital status, insurance status, T stage, N stage and histological type, and the prognostic nomogram established on the basis of these factors has high reliability.^[19]^ In a nomogram used to predict the overall postoperative survival of Chinese TNBC patients, among 336 patients, age, marital status, tumor site, grade, T stage and N stage were independent prognostic factors.^[26]^ Unmarried women are reported to be more likely to develop advanced breast cancer because having a spouse may be a factor in supporting early detection of malignancy.^[27,28]^ In our study, being married was a relevant risk factor for developing distant metastasis, whereas being unmarried was a related prognostic risk factor after having distant metastasis.

Tissue type, T stage, N stage, and tumor size were shown to be relevant factors for distant metastasis in TNBC. Wang J et al^[29]^ reported that in a nomogram for TNBC lung metastasis, age, tumor size, T stage and N stage were independent risk factors for LM in patients with TNBC. The histological type, marital status, preoperative surgery, chemotherapy, bone metastasis, brain metastases, and LM were found to be independent prognostic factors in patients with TNBC. Contrasting with our study, histological type correlated with distant metastasis but not with prognosis; this discrepancy may stem from patient selection bias, as the referenced literature exclusively focused on lung metastasis cohort.

Recent studies have shown that, compared with other subtypes of breast cancer, TNBC has a greater sensitivity to NACT and a stronger correlation with pCR and patient prognosis.^[30]^ A recent meta-analysis systematically reviewed published randomized controlled trials on NAT for TNBC, revealing a significant correlation between pC and event-free survival, as well as a favorable association between pCR and OS.^[31]^ Our study results indicate that patients who achieve CR after NAT have a better prognosis, but more robust evidence is needed to consider pCR as the optimal surrogate endpoint during neoadjuvant treatment. Further clinical studies are needed to explore appropriate trial endpoints.

Previous studies have shown that chemotherapy and surgery can significantly improve patient outcomes.^[32]^ At present, chemotherapy remains the standard treatment for patients with TNBC.^[33]^ Previous studies have shown that patients can benefit from surgery despite metastasis to distant organs.^[34,35]^ Our prognostic nomogram indicates that surgery and chemotherapy are beneficial for the survival of patients with TNBC accompanied by distant metastasis. Therefore, identifying independent prognostic factors may help in identifying high-risk patients and establishing a specific surveillance program.

However, our study has several limitations. The SEER database contains data on the first diagnosis of the disease; therefore, metastases occurring in late stages cannot be recorded. Furthermore, this is a retrospective study with a large sample size; therefore, selection bias is inevitable. A prognostic nomogram was constructed and validated at a single institution, which may partly influence its clinical applicability. Therefore, further calibration of the nomogram is needed in future studies.

## 5. Conclusion

This study identified marital status, histological type, N stage, T stage, surgery, radiotherapy, and tumor size as independent risk factors for distant metastasis in TNBC patients. Additionally, marital status, surgery, chemotherapy and NAT were found to be independent prognostic factors for patients with distant metastasis. We developed 2 nomograms utilizing diverse indicators to directly assess patient risk. The impressive performance of our nomograms in both the training and validation cohorts holds promise for assisting doctors in determining patient prognosis and devising personalized treatment plans.

## Author contributions

**Conceptualization:** Hongguo Guo, Wanling Lu.

**Data curation:** Cai Cheng, Jun Liu, Shangzhen Yang.

**Formal analysis:** Hongguo Guo, Song Qiao, Cai Cheng, Jun Liu.

**Investigation:** Jun Liu.

**Methodology:** Hongguo Guo.

**Software:** Hongguo Guo, Song Qiao.

**Validation:** Song Qiao, Shangzhen Yang.

**Visualization:** Cai Cheng, Shangzhen Yang.

**Writing – original draft:** Hongguo Guo, Jun Liu, Shangzhen Yang.

**Writing – review & editing:** Wanling Lu.
